# Concurrent ciliary body detachment in patients presenting with serous choroidal detachment following glaucoma surgery

**DOI:** 10.1007/s10792-024-03219-1

**Published:** 2024-06-26

**Authors:** Edward Barayev, Orly Gal-Or, Assaf Gershoni, Amir Hadayer, David Barash, Irit Bahar, Noa Geffen, Alon Zahavi

**Affiliations:** 1 Sackler Faculty of Medicine, Tel Aviv University, Tel Aviv, Israel; 2 Department of Ophthalmology, Rabin Medical Center – Beilinson Hospital, 39 Jabotinsky St., 4941492 Petach Tikva, Israel; 3 Laboratory of Eye Research, Felsenstein Medical Research Center, Petach Tikva, Israel

**Keywords:** Choroidal detachment, Ciliary body detachment, Glaucoma surgery, Hypotony, Ultrasound biomicroscopy

## Abstract

**Purpose:**

To examine the rate of ciliary body detachment in patients with choroidal detachment following glaucoma surgery and its effect on the clinical course, management, and prognosis.

**Methods:**

A prospective observational case-series study. Patients with choroidal detachment following glaucoma surgery in 2018–2019 were included. All underwent complete ophthalmological examination and ultrasound biomicroscopy for evaluation of the presence and extent of ciliary body detachment. Follow-up examinations including ultrasound biomicroscopy scans were performed at 1 week, 1 month, 3 months, and 6 months.

**Results:**

Eight patients (8 eyes) were enrolled, 4 male and 4 female, of mean age 72 years (range 60–83). Five patients underwent trabeculectomy with mitomycin C (0.02%), which was combined with phacoemulsification cataract extraction in one; two underwent Ahmed glaucoma valve implantations, and one underwent ab-interno Xen45 gel stent implantation with mitomycin C (0.02%). The mean intraocular pressure was 26.0 ± 7.65 mmHg preoperatively, dropping to 6.9 ± 2.64 mmHg on first postoperative day one. Mean time from surgery to diagnosis of choroidal detachment was 11.6 ± 5.73 days. Ciliary body detachment was identified by ultrasound biomicroscopy in all patients, ranging between one and four quadrants. All patients were treated with topical steroids and cycloplegics; three (37.5%) received oral steroids. No surgical intervention for the choroidal or ciliary body detachments was indicated.

**Conclusions:**

In this real-world prospective study, concurrent ciliary body detachment was identified in all patients who presented with choroidal detachment following glaucoma surgery. This observation may deepen our understanding of the mechanism underlying the hypotony that is often seen after glaucoma surgery.

## Introduction

The choroid is attached to the sclera in the normal eye. It may be displaced by serous fluid or blood accumulating inside the potential space between the uvea and sclera, leading to choroidal detachment [1]. Serous choroidal detachments are typically associated with low intraocular pressure (IOP) or ocular hypotony, and most commonly occur after eye surgery. Inflammation, trauma, malignancy, and certain medications can also cause serous choroidal detachment [2–8].

Like the choroid, the ciliary body may also detach, resulting in hypotony caused by reduced aqueous humor production. The simultaneous detachment of the choroid and the ciliary body (ciliochoroidal detachment), may result in ocular hypotony and compromise visual acuity [7, 9].

Ultrasound biomicroscopy (UBM) is a well-established noninvasive anterior-segment imaging method for diagnosing the presence and extension of choroidal and ciliary body detachments, as well as for the assessment of their clinical course [9–12].

The aim of the present study was to examine the rate of ciliary body detachment in patients with choroidal detachment following glaucoma surgery and its effect on the clinical course, management, and prognosis.

## Methods

A prospective observational case-series study was conducted in a single tertiary university-affiliated medical center. The study was approved by the local Institutional Ethics Committee of the Rabin Medical Center, Petach-Tiqva, and adhered to the tenets of the Declaration of Helsinki. Written informed consent was obtained from all participants.

The study included patients over the age of 18 years who underwent glaucoma surgery and were subsequently diagnosed with choroidal detachment. Choroidal detachment was defined as an elevation of the retina and choroid on both fundoscopic and B-scan ultrasound examinations. The presence and extent of ciliary body detachment was assessed by ultrasound biomicroscopy (UBM) using both probes of the Eye Cubed Ophthalmic Ultrasound System (Ellex/Innovative Imaging, Inc, Adelaide, Australia).

Excluded from the study were patients who were unable to undergo intraocular pressure (IOP) measurements or ophthalmic or UBM examinations, in addition to patients who were diagnosed with choroidal detachment prior to glaucoma surgery or had a history of intraocular surgery other than uncomplicated phacoemulsification or in whom the choroidal detachment had other possible causes such as ocular malignancy, ocular trauma, or intraocular inflammation.

Follow-up examinations were scheduled on 1 day, 1 week, and 1, 3 and 6 months after surgery. Patients with persistent detachments were followed until complete resolution.

Data were collected for each patient as follows: age and gender, systemic medical history, ocular history, glaucoma type, ocular medications, preoperative IOP, visual acuity, and type of glaucoma surgery. Each visit included an assessment of best corrected visual acuity (BCVA), measurement of IOP with a Goldmann applanation tonometer, comprehensive slit-lamp examination, and UBM examination performed by one of two UBM specialists (O.G.O. and D.B.(. The specialists evaluated the choroidal detachment with particular attention to its location, extent, and height, and presence of concurrent ciliary body detachment. The timing relative to glaucoma surgery on which choroidal detachment was diagnosed, its clinical and ultrasonic appearance, and its clinical course, management, and outcomes were assessed.

Postsurgical treatment was administered at the discretion of the surgeon and tailored to the patient. It generally included topical cycloplegic agents, antibiotic drops, as well as topical and/or oral steroids. The duration of treatment varied according to the clinical course.

## Statistical analysis

Statistical analysis was performed using IBM SPSS Statistics for Windows, version 24 (IBM Corp., Armonk, N.Y., USA). Continuous variables were summarized by mean and standard deviation. Pearson test was used to analyze correlations. *P* values were two-sided and considered statistically significant at < 0.05.

## Results

Eight consecutive patients (8 eyes) with choroidal detachment following glaucoma surgery were enrolled in the study. Their demographic and clinical characteristics are shown in Table 1. The study included four male and four female patients with a mean age of 72 years (range 60–83 years). Diagnoses were pseudoexfoliation glaucoma in four patients, neovascular glaucoma following central retinal vein occlusion in one patient, closed angle glaucoma in one, and glaucoma following an ocular trauma necessitating penetrating keratoplasty in one. The last patient was a steroid responder who was treated orally for adult-onset Still’s disease.

**Table 1:** Demographic and ciliary body detachment characteristics of study participants

#	Sex	Age (years)	Glaucoma type	Surgery	Follow-up period (years)	CBD duration (months)	Time from surgery to CCD (days)	Pre-op IOP (mmHg)	Post-op IOP (mmHg)	Change in IOP (mmHg)	CBD extent (degrees)	VA before surgery	VA at CCD diagnosis	VA at last follow-up	Treatment
1	F	83	Steroid responder	AGV	3.05	15.30	8	20	3	17	180	20/63	1/100	1/100	Topical & Oral Steroids, Cycloplegics
2	F	73	CACG	Trab + Phaco	2.71	6.50	10	27	10	17	270	20/160	3/40	20/30	Topical Steroids, Cycloplegics
3	M	63	PXFG	XEN	2.42	5.97	8	26	10	16	360	20/25	20/50	20/20	Topical Steroids, Cycloplegics
4	F	83	PXFG	Trab	2.72	0.87	13	25	4	21	270	20/40	20/40	20/50	Topical & Oral Steroids, Cycloplegics
5	M	60	Traumatic	AGV	2.08	1.37	7	18	7	11	360	3/80	3/60	20/100	Topical & Oral Steroids, Cycloplegics
6	M	68	PXFG	Trab	1.64	2.30	8	38	8	30	270	20/22	20/40	1/160	Topical Steroids, Cycloplegics
7	M	71	PXFG	Trab	2.94	0.37	24	18	8	10	90	20/30	20/400	20/50	Topical Steroids, Cycloplegics
8	F	76	NVG	Trab	3.24	1.57	15	36	5	31	360	20/50	20/400	20/50	Topical Steroids, Cycloplegics
Mean ± SD		72.13 ± 8.46			2.60 ± 0.53	4.28 ± 5.01	11.62 ± 5.73	26.00 ± 7.65	6.88 ± 2.64	19.13 ± 7.85	270.00 ± 103.92				

AGV, Ahmed glaucoma valve; CACG, chronic angle closure glaucoma; CBD, ciliary body detachment; CCD, ciliochoroidal detachment; IOP, iptraocular pressure; Phaco, phacoemulsification; PXFG, pseudoexfoliative glaucoma; Trab, trabeculectomy; VA, visual acuity XEN, XEN 45 gel stent

Five patients underwent trabeculectomy with mitomycin C (0.02%), which was combined phacoemulsification cataract surgery in one; two patients underwent Ahmed glaucoma valve (New World Medical, Rancho Cucamonga, CA, USA) implantation; and one underwent ab-interno Xen45 gel stent (Allergan Inc., Irvine, CA, USA) implantation with mitomycin C (0.02%). The mean IOP was 26.0 ± 7.65 mmHg preoperatively and 6.9 ± 2.64 mmHg on postoperative day 1, for a mean decrease of 19.1 ± 7.85 mmHg.

**Fig. 1:**
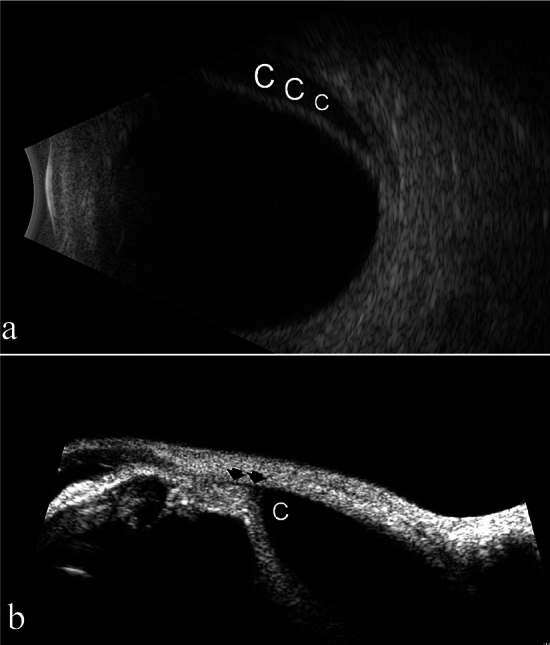
Ciliochoroidal detachment in an 83-year-old patient 3 months after Ahmad glaucoma valve surgery, as demonstrated by B-scan ultrasonography (**a**) and ultrasound biomicroscopy (**b**). C = choroidal detachment. Arrows indicate the ciliary body detachment

The mean time from surgery to the diagnosis of choroidal detachment was 11.6 ± 5.73 days. Ciliary body detachment was identified by UBM in all patients, ranging between one and four quadrants. Findings on exemplary B-scan and UBM examinations of ciliochoroidal detachment in a representative case are presented in Fig. 1a and b, respectively. There was no statistically significant correlation between the preoperative and postoperative IOP measurements, or between the difference between the two measurements and the extent of ciliary body detachment (Table 2).

**Table 2:** Correlations between extent of ciliary body detachment and IOP measurements in the study participants

Variable	r	*P*
Preoperative IOP	0.419	0.301
Postoperative IOP	0.152	0.720
Change in IOP	0.358	0.384

IOP, intraocular pressure. r, Pearson correlation coefficient

Ciliochoroidal detachment in all patients was treated with topical steroids (0.1% dexamethasone phosphate every 6 h to every hour) and cycloplegics (cyclopentolate 1% 3 times/day); 3 patients (37.5%) received additional oral steroids. All patients were managed conservatively, and none required surgical intervention. The mean time to full resolution of the detachment was 4.3 ± 5.01 months. With the exclusion of patient 1 in whom the duration of ciliary body detachment was abnormally prolonged, the mean time of treatment with ciliochoroidal detachment drops was 2.7 ± 2.48 months. None of the patients experienced complications related to ciliary body detachment. Patients 1 and 6 had decreased visual acuity at the last follow-up which was attributed to endophthalmitis and mature cataract, respectively.

## Discussion

This is the first prospective study to assess the prevalence of ciliary body detachment in patients with choroidal detachment following glaucoma surgery. We found that ciliary body detachment coexisted with choroidal detachment in all patients.

In the normal eye, the suprachoroidal space is essentially nonexistent owing to the close apposition of the highly vascular and permeable choroid to the sclera [13]. In pathologic circumstances, due to disruption of ocular fluid dynamics, fluids can accumulate in this potential space. Low IOP after glaucoma surgery is the most common causative factor of serous choroidal effusions, which are composed of transudate fluids that build up as a consequence of increased transmural pressure across the capillaries [14–16]. Although overfiltration in the immediate postoperative period may explain the choroidal detachment, another possible contributing factor is impaired aqueous humor production, previously described to occur following glaucoma surgery. This may be due to antimetabolite toxicity to the ciliary body, postoperative inflammation, and/or iatrogenic cyclodialysis cleft formation [10, 17, 18]. Nonetheless, we theorize that not only did these factors facilitate the ciliary body shutdown, they also led to detachment of the ciliary body itself. While optical coherence tomography may be used for ciliary body imaging, the gold standard is UBM, which was used in our study [19, 20]. Our finding of concurrent ciliary body detachment in all cases of choroidal detachment sheds more light on the pathophysiology of the disease and the vicious cycle that transpires after choroidal detachment occurs. As the ciliary processes are the main site of aqueous humor production [13], ciliary body detachment may have an added detrimental effect on aqueous production and may thereby play an important role in postoperative hypotony.

A large persistent choroidal effusion may have immediate and long-lasting vision-threatening sequelae, particularly when it is associated with hypotony, maculopathy, or serous retinal detachment [2, 17]. However, most serous choroidal effusions follow a benign course as they may be asymptomatic and resolve spontaneously without treatment when the IOP gradually increases postoperatively [21]. If treatment is warranted, topical steroids are usually prescribed in an effort to increase IOP and reduce inflammation. Long-acting topical cycloplegics may be given to deepen the anterior chamber and prevent anterior synechiae and corneal endothelial cell loss [22]. When topical treatment is not sufficient, systemic steroids may be used [21, 23]. Surgical intervention is indicated when the choroidal detachment is longstanding or causes complications that can lead to loss of vision [24]. In our cohort, treatment was left to the treating physician’s discretion. All patients received topical steroids and cycloplegic agents, and some elected to add systemic steroids. Most maintained their visual acuity following resolution of the ciliary body detachment, even when it was longstanding. In the two patients who lost two or more Snellen lines of BCVA, a mature cataract and status post endophthalmitis were the presumed causes.

The major limitations of this study are the small number of patients examined and the heterogeneity of glaucoma types. Moreover, the various types of glaucoma surgeries performed in our cohort could have had different implications for the occurrence of ciliary body detachment. The strengths of the study are the prospective design and the long duration of follow-up.

To conclude, in this real-world prospective study, we found concurrent ciliary body detachment in all patients who presented with choroidal detachment following glaucoma surgery. This observation may deepen our understanding of the postoperative mechanism underlying hypotony. We found that all detachments resolved with no need for surgical intervention. Larger prospective studies are warranted.

## Data Availability

No datasets were generated or analysed during the current study.

## References

[1] American Academy of Ophthalmology. Basic and Clinical Science Course. Section 12: Retina and Vitreous 2019–2020. San Francisco, CA: USA, p 379

[2] Bellows AR, Chylack LT Jr, Hutchinson BT (1981) Choroidal detachment. Clinical manifestation, therapy and mechanism of formation. Ophthalmology 88:1107–1115 7335316

[3] Arnalich-Montiel F, Ruiz-Casas D, Munoz-Negrete F, Rebolleda G (2015) Inadvertent cyclodialysis cleft and annular ciliochoroidal detachment after hyperopic phakic intraocular lens implantation and prophylactic surgical iridectomy. J Cataract Refract Surg 41:2319–2322. 10.1016/j.jcrs.2015.09.010 26703309 10.1016/j.jcrs.2015.09.010

[4] Brubaker RF, Pederson JE (1983) Ciliochoroidal detachment. Surv Ophthalmol 27:281–289. 10.1016/0039-6257(83)90228-x 6407132 10.1016/0039-6257(83)90228-x

[5] Tarantola RM, Folk JC, Shah SS, Boldt HC, Abràmoff MD, Russell SR, Mahajan VB (2011) Intraoperative choroidal detachment during 23-gauge vitrectomy. Retina 31:893–901. 10.1097/IAE.0b013e3181f4429b 21273944 10.1097/IAE.0b013e3181f4429b

[6] Yamane S, Inoue M, Arakawa A, Kadonosono K (2012) Early postoperative hypotony and ciliochoroidal detachment after microincision vitrectomy surgery. Am J Ophthalmol 153:1099-1103.e1. 10.1016/j.ajo.2011.11.001 22310085 10.1016/j.ajo.2011.11.001

[7] Yang JG, Yao GM, Li SP, Xiao-Huawang RBC (2011) Surgical treatment for 42 patients with traumatic annular ciliochoroidal detachment. Int J Ophthalmol 4:81–84. 10.3980/j.issn.2222-3959.2011.01.19 22553616 10.3980/j.issn.2222-3959.2011.01.19 PMC3340687

[8] Elagouz M, Stanescu-Segall D, Jackson TL (2010) Uveal effusion syndrome. Surv Ophthalmol 55:134–145. 10.1016/j.survophthal.2009.05.003 20159229 10.1016/j.survophthal.2009.05.003

[9] Alibet Y, Levytska G, Umanets N, Pasyechnikova N, Henrich PB (2017) Ciliary body thickness changes after preoperative anti-inflammatory treatment in rhegmatogenous retinal detachment complicated by choroidal detachment. Graefes Arch Clin Exp Ophthalmol 255:1503–1508. 10.1007/s00417-017-3673-2 28493087 10.1007/s00417-017-3673-2 PMC5541079

[10] Martínez-Belló C, Capeáns C, Sánchez-Salorio M (1999) Ultrasound biomicroscopy in the diagnosis of supraciliochoroidal fluid after trabeculectomy. Am J Ophthalmol 128:372–375. 10.1016/s0002-9394(99)00118-x 10511041 10.1016/s0002-9394(99)00118-x

[11] Wada S, Kohno T, Yanagihara N, Hirabayashi M, Tabuchi H, Shiraki K, Miki T (2002) Ultrasound biomicroscopic study of ciliary body changes in the post-treatment phase of Vogt-Koyanagi-Harada disease. Br J Ophthalmol 86:1374–1379. 10.1136/bjo.86.12.1374 12446369 10.1136/bjo.86.12.1374 PMC1771437

[12] Yuki T, Kimura Y, Nanbu S, Kishi S, Shimizu K (1997) Ciliary body and choroidal detachment after laser photocoagulation for diabetic retinopathy. A high-frequency ultrasound study. Ophthalmology 104:1259–1264. 10.1016/s0161-6420(97)30149-3 9261312 10.1016/s0161-6420(97)30149-3

[13] American Academy of Ophthalmology. Basic and clinical science course. Section 10: Glaucoma. San Francisco: 2019–2020. p 13–16

[14] Jampel HD, Musch DC, Gillespie BW, Lichter PR, Wright MM, Guire KE, Collaborative Initial Glaucoma Treatment Study Group (2005) Perioperative complications of trabeculectomy in the collaborative initial glaucoma treatment study (CIGTS). Am J Ophthalmol 140:16–22. 10.1016/j.ajo.2005.02.013 15939389 10.1016/j.ajo.2005.02.013

[15] Haga A, Inatani M, Shobayashi K, Kojima S, Inoue T, Tanihara H (2013) Risk factors for choroidal detachment after trabeculectomy with mitomycin C. Clin Ophthalmol 7:1417–1421. 10.2147/OPTH.S46375 23874083 10.2147/OPTH.S46375 PMC3713998

[16] Iwasaki K, Kakimoto H, Arimura S, Takamura Y, Inatani M (2020) Prospective cohort study of risk factors for choroidal detachment after trabeculectomy. Int Ophthalmol 40:1077–1083. 10.1007/s10792-019-01267-6 31989350 10.1007/s10792-019-01267-6

[17] Wang Q, Thau A, Levin AV, Lee D (2019) Ocular hypotony: a comprehensive review. Surv Ophthalmol 64:619–638. 10.1016/j.survophthal.2019.04.006 31029581 10.1016/j.survophthal.2019.04.006

[18] Mietz H (1996) The toxicology of mitomycin C on the ciliary body. Curr Opin Ophthalmol 7:72–79 10163326 10.1097/00055735-199604000-00013

[19] Xinping Y, Weihua P, Mei R, Jia Q (2011) Supraciliochoroidal fluid incidence at the early stage after trabeculectomy: study with anterior segment optical coherence tomography. Curr Eye Res 36:818–823. 10.3109/02713683.2011.593724 21851168 10.3109/02713683.2011.593724

[20] Fernández-Vigo JI, Kudsieh B, Shi H, De-Pablo-Gómez-de-Liaño L, Fernández-Vigo JÁ, García-Feijóo J (2022) Diagnostic imaging of the ciliary body: technologies, outcomes, and future perspectives. Eur J Ophthalmol 32:75–88. 10.1177/11206721211031409 34233517 10.1177/11206721211031409

[21] Schrieber C, Liu Y (2015) Choroidal effusions after glaucoma surgery. Curr Opin Ophthalmol 26:134–142. 10.1097/ICU.0000000000000131 25643198 10.1097/ICU.0000000000000131

[22] de Barros DS, Navarro JB, Mantravadi AV, Siam GA, Gheith ME, Tittler EH, Baez KA, Martinez SM, Spaeth GL (2009) The early flat anterior chamber after trabeculectomy: a randomized, prospective study of 3 methods of management. J Glaucoma 18:13–20. 10.1097/IJG.0b013e31816f7647 19142129 10.1097/IJG.0b013e31816f7647

[23] Ku WC, Lin YH, Chuang LH, Yang KJ (2005) Choroidal detachment after filtering surgery. Chang Gung Med J 28:151–158 15945321

[24] WuDunn D, Ryser D, Cantor LB (2005) Surgical drainage of choroidal effusions following glaucoma surgery. J Glaucoma 14:103–108. 10.1097/01.ijg.0000146370.28625.fc 15741809 10.1097/01.ijg.0000146370.28625.fc

